# Segmental testicular infarction: A case report of a rare condition

**DOI:** 10.1016/j.eucr.2022.102115

**Published:** 2022-05-13

**Authors:** Bilel Saidani, Marouene Chakroun, Saadi Ahmed, Mokadem Seif, Mohamed Riadh Ben Slama, Chebil Mohamed

**Affiliations:** University of Tunis El Manar, Faculty of Medicine of Tunis, Charles Nicolle Hospital, Urology Department, Tunis, Tunisia

**Keywords:** Testicular infraction, Scrotal pain, Orchiectomy

## Abstract

Segmental testicular infarction(STI) is a rare unknown condition. Clinical presentation is non-specific and management is unconsensual. We report clinical and surgical management of a segmental testicular infarction. A 18-years old adult presented to emergency with acute right testicular pain. The only triggering factor was a stressful situation. Examination revealed a slightly swollen, non-ascending testicle with no inflammatory signs. We suspected spermatic –cord torsion(SCT), we decided to explore it surgically. Exploration showed a well-demarcated infarcted inferior pole of right testicle. Partial orchiectomy was performed. STI is a rare entity often clinically confused with SCT. Diagnosis is based on intraoperative findings.

## 1 Introduction

Segmental testicular infarction is a rare condition and remains unknown to clinicians.^1^ The clinical presentation is unspecific and is similar in patients who consult in emergency for acute scrotal pain.^1^ Imaging by scrotal ultrasound or at best by MRI confirms the diagnosis .^1^^,^^2^ The data available remains poor and only few cases are reported in literature.^1^^,^^2^

We report the clinical and radiological data and surgical management of a case of segmental testicular infarction treated in our department.

## 2 Case presentation

**Fig. 1:**
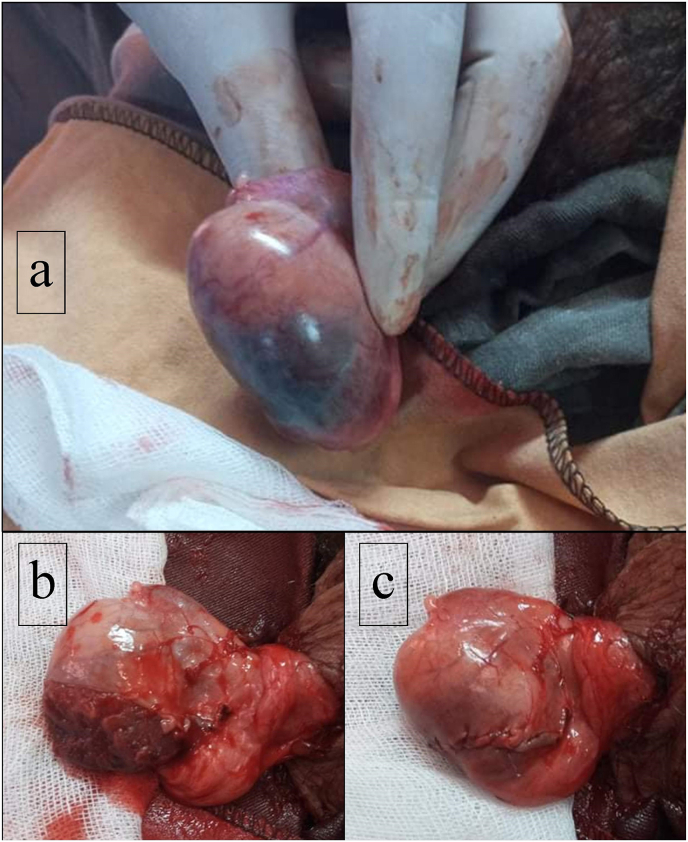
Surgical exploration of the testis: a: Well-demarcated infarcted appearance of the lower pole of the right testicle. b: Well-demarcated infarcted appearance of the lower pole of the right testicle after opening of the albuginea. c: Final appearance after partial orchiectomy.

We report the case of a 27 year old patient, with no medical history, who consulted our emergency department for intense acute right testicular pain, rated at 8 on the numerical scale, radiating to the right inguinal region, associated with nausea. The only triggering factor revealed an acute stressful situation preceding the onset of the pain. Examination found no fever. Examination of the testicles revealed a slightly swollen right testicle, not ascended, with no inflammatory signs. There was no palpable testicular mass. The spermatic cord was painless and free. The cremasteric reflex was present. Given the clinical suspicion of a torsion of the spermatic cord in a young adult, we decided to explore the testicles surgically. Intraoperative exploration showed a right testicle of normal consistency and size, with no suspicious mass. There was no spermatic cord torsion. There was a well-demarcated infarcted appearance of the lower pole of the right testicle(Fig. 1a and b). A partial orchiectomy removing the infarcted pole was performed and the albuginea was closed with a 3/0 Vicryl suture(Fig. 1c). The patient was discharged 12 h after surgery. Thrombophilia evaluation returned without abnormalities. Pathological examination showed no malignant tissue of the infarcted testicular tissue.The patient was counselled to consult in case of infertility. Testosterone levels were not assessed as the left testis was normal.

## 3 Discussion

Segmental testicular infarction is a rare condition that has not enough been described in the literature.^3^^,^^4^ The idiopathic form is predominant.^1^ Pathophysiological reported hypotheses are: arterial or venous occlusion, inguinal hernia repair, hypercholesterolemia, orchi-epididymitis, antithrombin III or protein S deficiency, sickle cell anemia, neonatal trauma, malakopathy and lymphoid leukemia.^1^, ^2^, ^3^, ^4^ In our case, none of these factors were evident except for an acute stressful situation suggestive of the idiopathic form.

In our case, the infarction involved the lower pole of the testis. However, some authors have reported the superior pole of the testicle as the preferred site because of the poor supplementation of the anterior vascular network.^1^

The described age of predilection is between 16 and 35 years.^3^ The clinical presentation is often acute testicular pain without a traumatic or infectious context, as in our case.^3^ The clinical examination is non-specific and usually finds a normal spermatic cord.^1^^,^^3^

The clinical presentation may simulate a testicular tumour with a clinically suspicious mass with negative testicular markers leading to inguinal orchiectomy.^3^^,^^4^ In chronic forms, an atrophic testis is observed with retraction of the albuginea and loss of testicular volume.^1^ The diagnosis is often made anatomically after orchiectomy.^1^

Testicular Doppler ultrasonography is the first line of investigation, demonstrating an avascular area on Doppler.^2^^,^^3^ Distinguishing between infarction, tumor and testicular abscess is difficult on ultrasound.^4^

Scrotal MRI may be indicated in difficult cases and atypical sonographic forms.^2^^,^^3^ On T2 sequences, the lesion appears well limited and on hypo signal. Peripheral enhancement may be seen after injection of gadolinium and persists for 30 days after the infraction has occurred. During the follow-up MRI shows a progressive decrease in the size of the lesions, indicating the loss of testicular tissue.^5^

In our patient, the fear of missing a torsion of the spermatic cord led us to perform an emergency surgical exploration without carrying out radiological examinations. It should be noted that none of these radiological methods can eliminate a testicular tumor.^5^

For management, surgical exploration is the rule for some teams while others advocate pain management and clinical and ultrasound monitoring.^1^^,^^3^

Surgical exploration is ideally performed through an inguinal approach with first vascular clamping.^3^ Some teams advocate systematic orchiectomy, others encourage partial surgery in case of small lesions, not vascularized on Doppler with normal tumor markers.^1^^,^^2^ Partial orchiectomy is recommended because of the risk of recurrence, especially contralateral.^1^ Extemporaneous pathological examination may be useful before conservative treatment.^3^ Conservative treatment should be considered with a strong presumption of a benign lesion.^4^ In our patient we performed a partial orchiectomy and the pathological examination of the specimen denied the presence of malignant lesion.

## 4 Conclusion

Segmental testicular infarction is a rare entity often clinically confused with spermatic cord torsion. The diagnosis is based on intraoperative findings. Ultrasound and MRI are recommended to guide the diagnosis. Partial surgery may be proposed in the absence of malignant suspicion.

## Consent

Signed consent was obtained from the patient.

## Declaration of competing interest

The authors declare that there are no conflicts of interest regarding the publication of this article.
